# Pain Control by Proprioceptive and Exteroceptive Stimulation at the Trigeminal Level

**DOI:** 10.3389/fphys.2018.01037

**Published:** 2018-08-07

**Authors:** Claudio Zampino, Roberta Ficacci, Miriam Checcacci, Fabio Franciolini, Luigi Catacuzzeno

**Affiliations:** ^1^Department of Chemistry, Biology and Biotechnology, The University of Perugia, Perugia, Italy; ^2^Azienda Sanitaria Locale Roma 1, Rome, Italy

**Keywords:** gate control theory, dental pain, trigeminal system, exteroceptive and proprioceptive stimulation, mandibular extension

## Abstract

The Gate Control Theory of pain, published more than half a century ago to explain nociceptive modulation of peripheral sensory input, assumes inhibition of incoming nociceptive (pain) information produced by mechanical stimulation. To verify the presence of such a gate control mechanism at the level of the human trigeminal system, we evaluated the effects on pain sensation of a proprioceptive trigeminal stimulation induced by mandibular extension. We found that such a stimulation, applied for 7 min, was effective in increasing both the threshold and tolerance of tooth pain induced by electrical activation of dental nociceptors. Moreover the antinociceptive effect lasted for several minutes after the proprioceptive stimulus had ceased. We also tested whether an exteroceptive palatal stimulation superimposed on the proprioceptive stimulation would increase the effects on tooth pain perception of human volunteers. We observed that the exteroceptive stimulation significantly increased the antinociceptive effect induced by the sole proprioceptive stimulation. The physiological mechanisms and the possible implications of these observations are discussed.

## Introduction

Pain originates at the peripheral level and is transported to the central nervous system by sensory fibers (myelinated Aδ and unmyelinated C) called nociceptors. Within the spinal cord, nociceptors transmit the pain information to second order neurons that will in turn transport the message to more encephalic structures, such as the thalamus and the cortex.

Pain perception is quite subjective, due to several kinds of modulation by neurons of both the central and peripheral nervous system (Treede, 2016). A major modulatory mechanism is the so-called Gate Control Theory (GCT), hypothesized for the first time by Melzack and Wall (1962). This theory explains the finding that pain originating from a certain body area may be alleviated by tactile, non-nociceptive stimulation of the same region. An example of this modulation is represented by the beneficial effects of a massage on a painful body region. Notably, on the GCT lays the rationale of several currently used therapeutic analgesic strategies (Kuwahara and Ogawa, 2016). While the gate control has been widely demonstrated at the level of the body areas innervated by dorsal root ganglions, only few evidences have been obtained for this pain modulation at the level of the trigeminal system (Brunelli et al., 2001a,b). A clear evidence for the presence of a trigeminal gate control would surely contribute to new analgesic approaches to treat pain originating from the head and neck.

Among the many structures innervated by the trigeminal nerve, the dental pulp has a special place: It is thought to be innervated exclusively by nociceptors, as suggested by the finding that tooth stimulation produces pain as the only perception (Byers, 1984; Byers and Närhi, 1999), and all dental fibers originate from the trigeminal nerve. Trigeminal sensory terminals reaching the dental pulp are strictly associated to the blood vessels to form a compact neurovascular unit, of which only few ramifications reach the crown pulp and the dentin (Bergenholtz et al., 2010). Tooth nociceptors belong to unmyelinated (C), thinly myelinated (Aδ), and, to a small extent (about 7%), myelinated (Aβ) sensory neurons (Närhi et al., 1996; Ingle et al., 2008). It has been further shown that Aβ terminals respond to the movement of the dentin fluid (Dong et al., 1985) and have a relatively low activation threshold, as compared to C and Aδ fibers (Trowbridge, 1986; Figdor, 1994; Niharika et al., 2013).

The trigeminal nerve also innervates the temporomandibular joint (TMJ) and masticatory muscles. Terminal sensory fibers located within the TMJ respond to the movement of the condyle and meniscus, and encode the TMJ position. They include non-adapting Ruffini endings and rapidly adapting Pacinian corpuscles, reporting the TMJ angle and its variation, respectively. Masticatory muscles are instead innervated by muscle spindles sensitive to the fibers length (Malerba, 2017). The trigeminal nerve, with its nasopalatine branch, provides also a high density of palatal receptors, especially in the palatin rugae which is frequently stimulated by the tongue. More specifically, the region includes Meissner corpuscles and Ruffini endings (Halata and Baumann, 1999; Schmidt et al., 2009; di Vico et al., 2013). For their anatomy and functional projections, both these trigeminal nerve branches could be involved in the gate control mechanism of pain of the oral district, and their activity could be associated with nociceptive modulation and pain perception.

In this paper we aim to provide evidence for this notion. To this end we evaluated dental pain in the absence and presence of a proprioceptive stimulation provided by a mandibular extension. We then verified whether the addition of an exteroceptive stimulus from the palatal area could increase the modulatory effect of the proprioceptive stimulation on pain sensation. Our data indicate a significant and additive attenuation of the pain perception following both kinds of non-nociceptive stimulation.

## Materials and Methods

In this study we included 296 human healthy subjects of 22–45 years of age, both males (*n* = 135) and females (*n* = 161). We did not discuss with them on the possible efficacy of the stimulation. The pain perception was evaluated by electrical stimulation of the incisor tooth by using a device routinely used to evaluate the vitality of the dental pulp (Vitality Scanner 2006, Analytic-Technology Corp, Redmond, WA, United States). Only subjects with healthy incisors were included. Incisors were dried and a fluoride gel was applied to improve the contact with the electrical probe. The intensity of the electrical stimulus was increased at a constant rate while an intensity indicator (from 0 to 80) was continuously monitored. The subject was asked to indicate with the right/left hand the moment of first perception of tooth pain, and the corresponding intensity value was taken as a measure of the pain perception threshold (PPT). The intensity of the electrical stimulus was then gradually increased until the subject instinctively interrupted the contact with the probe when the pain was too strong to be tolerated. At this point, the corresponding intensity value was taken as a measure of the pain tolerance level (PTL). A similar use of the vitality scanner has been already reported (Gerschman and Giebartowski, 1991).

**FIGURE 1:**
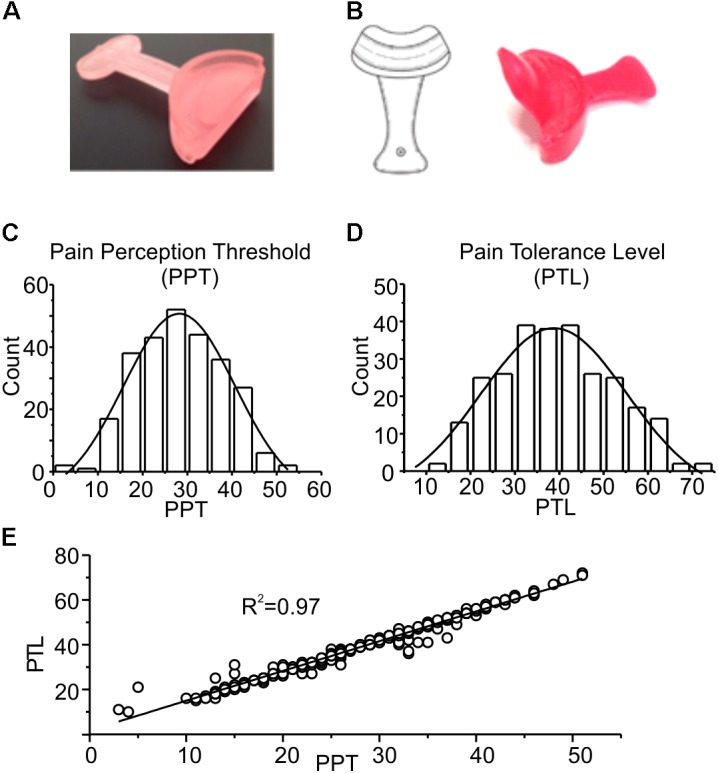
**(A)** Picture of the device used for mandibular extension in the trigeminal stimulation tests (proprioceptive type). The device consists of a 10 or 5-mm thickness to be placed in the mouth at the level of the incisors, having a width ranging from canine to canine. **(B)** Neurophysiological Stimulation Device (NSD) (Ficacci, 2014): an endoral neurophysiological stimulation having a half-moon shape, with a 10 or 5-mm thickness (trigeminal self-stimulation element for proprioceptive mandibular extension) similar to that described in **(A)** to be positioned in the anterior portion of the oral cavity, from canine to canine, between the upper and lower dental arches. The oblique fin of about 2–3 cm has the function to stimulate the palatine area. **(C,D)** Frequency distributions of PPT and PTL values. The experimental data were fitted with normal distributions (solid lines), with mean and standard deviation of 28.2 ± 5.0 and 38.8 ± 5.7, respectively. **(E)** Plot showing the strong linear correlation between the two parameters, with a Pearson coefficient of 0.97. The line is obtained from the fitting of the experimental data.

Subjects were in turn invited to enter the testing room, and after 2 min a first evaluation of the PPT and PTL was performed. After one more minute subjects were engaged in one of the following protocols: (1) control group (CTRL), on which no stimulation was applied (*n* = 112); (2) PROP group, on which a 7 min proprioceptive stimulation performed by imposing a mandibular extension of either 0.5 or 1 cm with the device shown in **Figure 1A** was applied (*n* = 128); (3) PROP+EXT group, on which a 7 min proprioceptive stimulation plus an exteroceptive stimulation (with 0.5 or 1 cm extension) was applied (*n* = 56); in this case a device similar to the previous one, but with an additional extension that mechanically stimulates the palate was used (**Figure 1B**). PPT and PTL were evaluated at different times during and after the stimulation, as described in the Results section.

## Results

**FIGURE 2:**
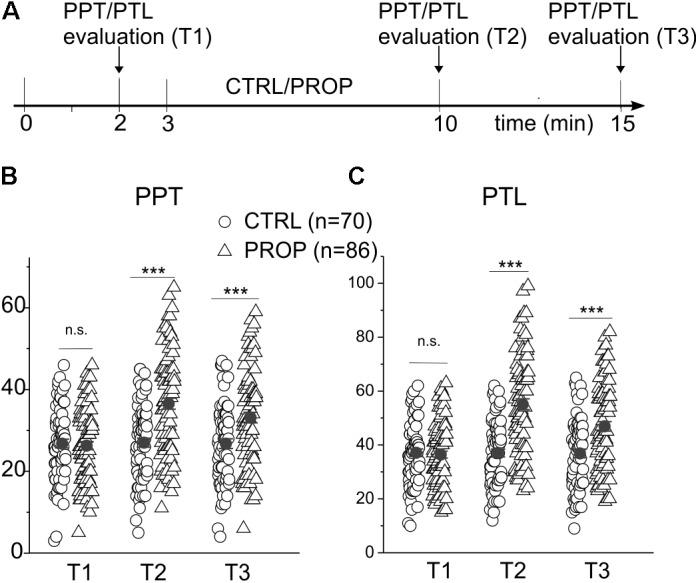
**(A)** Scheme of the experimental protocol used in this experiment. The participants were subject to a first test for the evaluation of the pain perception threshold (PPT) and pain tolerance level (PTL), 2 min after entering the testing room (min 2). After a further minute of rest (min 3) the subjects spent the next 7 min in one of the following conditions: (I) at rest without any auto-stimulation, and closed mouth (CTRL); (II) proprioceptive stimulation (PROP) with the device shown in **Figure 1A**, using a mandibular extension of 1 cm. Subsequently, at 0 (T2) and 5 min (T3) from the end of the stimulation, the PPT and PTL were recalculated. **(B)** Plot of the PPTs measured in CTRL subjects (circles) and in subjects undergoing a PROP stimulation (triangles) at the three different times. Also plotted is the mean ± SE of each group of the data (closed circles). **(C)** Same analysis made in **(B)** but on PTL values. ^∗∗∗^, *t*-test, *p* < 0.001; n.s., *t*-test, *p* > 0.05.

**FIGURE 3:**
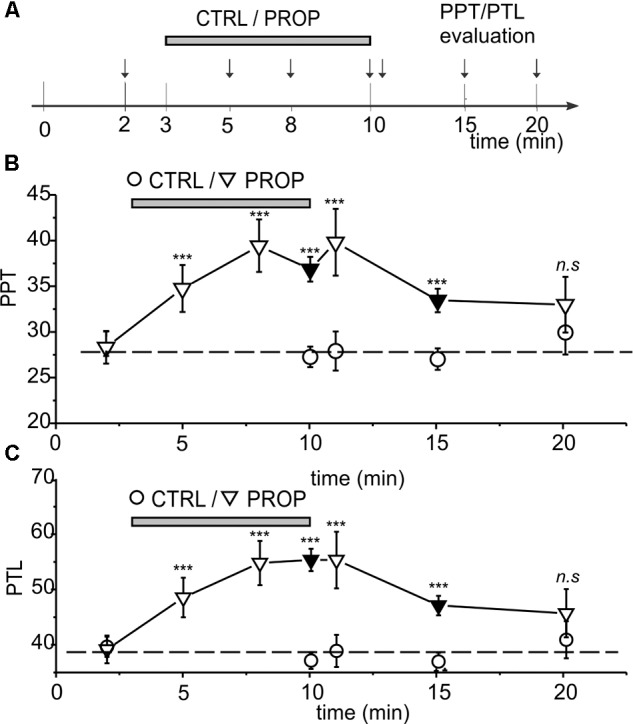
**(A)** Scheme showing all the time points were the PPT and PPT were assessed, in the three different experiments performed. **(A,B)** Time course of the effects of proprioceptive stimulation (PROP, 1 cm extension) on PPT **(B)** and PTL **(C)**. The circles represent control subjects (CTRL), while the triangles represent treated subjects (PROP). The data are from three separate experiments, as described in Methods. Black symbols refer to data already presented in **Figure 2**. ^∗∗∗^, *t*-test, *p* < 0.001; n.s., *t*-test, *p* > 0.05.

**Figures 1C–E** show a statistical analysis of the PPT and PTL values assessed at the beginning of the experiment in all subjects included in this study (*n* = 296). Both parameters are normally distributed, with mean and standard deviations of 28.2 ± 5.0 for PPT, and 38.8 ± 5.7 for PTL, respectively, as indicated by the χ^2^ test (χ^2^ = 18.1, *df* = 10, *p* > 0.05 for PPT and χ^2^ = 16.7, *df* = 12, *p* > 0.1 for PLT). **Figure 1E** shows a strong linear correlation between the two parameters, with a Pearson coefficient of 0.97. These data demonstrate that the assessed parameters are statistically uniform, and all subjects included in the study belong to the same population. **Figure 2** evaluates the effects of 7 min proprioceptive stimulation, attained via mandibular extension (1 cm) with the device shown in **Figure 1A**, by comparing the PPT and PTL values of 86 individuals subject to this protocol with those of 70 unstimulated individuals (i.e., not subject to any type of sensory stimulation of the mouth, CTRL). To avoid excessive strain to the enrolled subjects, in this experiment the assessment of the PPT and PTL was performed only at three time points: at the beginning of the experiment (T1, min 2), after 7 min of proprioceptive stimulation (PROP subjects), or rest (CTRL subjects) (T2, min 10), and after 5 min from the removal of the stimulus (T3, min 15) (**Figure 2A**). The results show that 7 min of proprioceptive stimulation increases both the PPT and PTL values by about 37 and 57%, respectively, as compared to control values (**Figures 2B,C**, triangles). This effect is most likely to be attributed to the proprioceptive stimulation, since in control subjects the PPT and PTL values remain approximately constant during the overall experiment (**Figures 2B,C**, circles). Notably, the analgesic effect of the proprioceptive stimulation can be clearly appreciated also after 5 min from the termination of the stimulation (**Figures 2B,C**, triangles), at which time the PPT and PTL values are still about 22 and 30% above the control values. In order to better evaluate the kinetics of appearance and disappearance of the effect following the termination of the proprioceptive stimulus, we performed additional experiments in which the PPT and PTL values were assessed either after 1 and 10 min from the termination of the stimulation or at 2 and 5 min from the beginning of the stimulation to assess the time course of the effect. The resulting data were then pooled with those of **Figure 2** to obtain the time course displayed in **Figure 3** which shows that: (1) in control (unstimulated) subjects PPT and PTL remained constant for the entire duration of the experiment, and had consistent values in the three different experiments; (2) in the subjects that performed the proprioceptive stimulation there was a gradual increase in the PPT and PTL values during the stimulation, and a slow disappearance of the effect starting from the end of the stimulation, that was almost complete after 10 min; (3) the time courses of the appearance and disappearance of the effect were very similar for the PPT and PTL.

**FIGURE 4:**
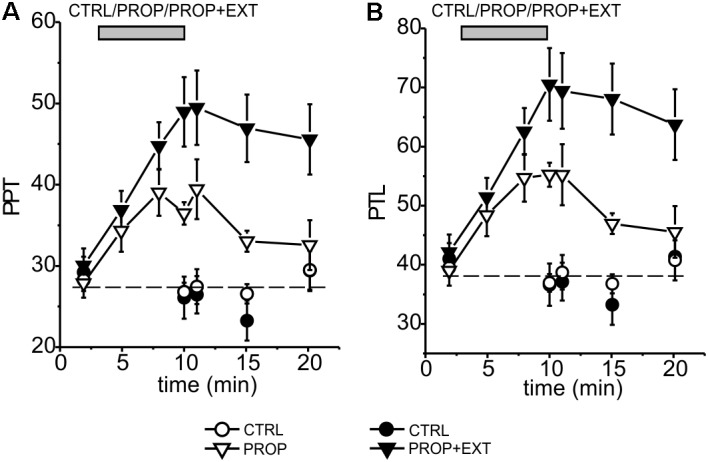
Time course of the mean effects promoted by proprioceptive stimulation (PROP, white triangles) or proprioceptive plus exteroceptive stimulation (PROP+EXT, black triangles) on the PPT **(A)** and PTL **(B)**. The circles represent control subjects (CTRL), the data come from six different experiments using a 1 cm high device for proprioceptive stimulation, and carried out as described in Methods.

We then verified whether the addition of an exteroceptive stimulus, consisting in the application of a mechanical pressure to the palatal area, could increase the antinociceptive effect of the proprioceptive stimulation. In these experiments we applied the proprioceptive and exteroceptive stimuli simultaneously by using the intraoral device illustrated in **Figure 1B** and detailed in Methods. Using this newly designed device, we repeated the above experiments already performed to test the sole proprioceptive stimulation. The results obtained are summarized in the time courses for the PPT and PTL shown in **Figure 4**, where also the already shown time courses for the sole proprioceptive stimulation are shown in order to facilitate the comparison. It is evident that: (i) the exteroceptive stimulation significantly increases the effect obtained with the sole proprioceptive stimulation, with the PPT and PTL being increased by the stimulation almost twice as much at the end of the stimulation period; (ii) the time course of the increase in the analgesic effect appears slower in the presence of the exteroceptive stimulation, so that the 7 min protocol does not allow to reach the maximal effect; (iii) also the time course of the disappearance of the effect after the end of the stimulation appears slower, with the result that at 10 min from the end of the stimulation a significant effect could still be observed for the proprioceptive plus exteroceptive stimulation, but not for the sole proprioceptive stimulation.

**FIGURE 5:**
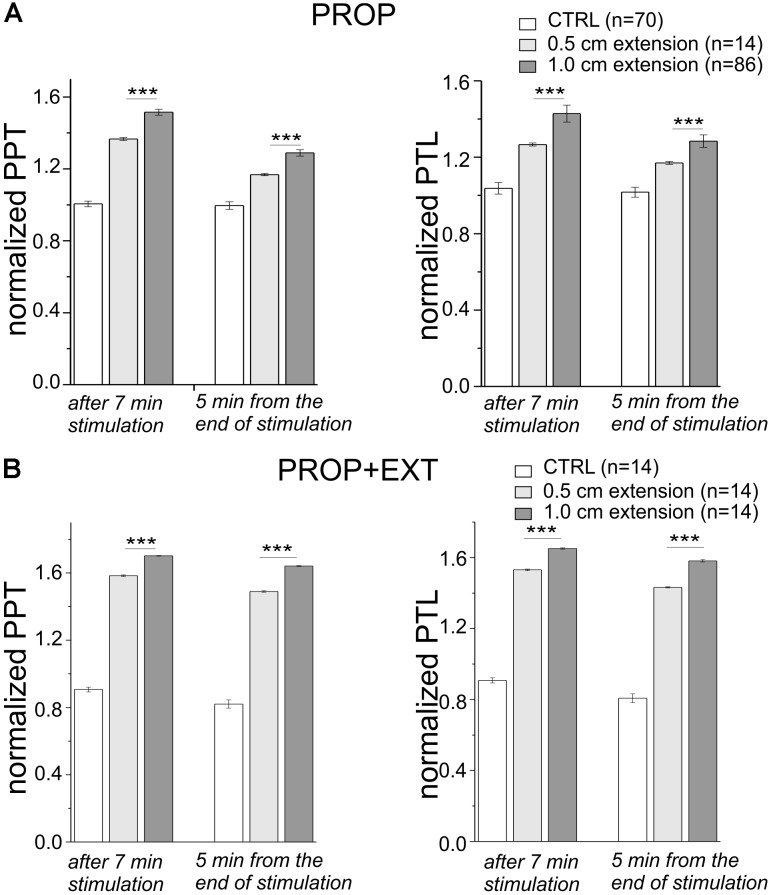
**(A,B)** Plot of the mean PPT (left) and PTL (right) taken after 7 min of stimulation and 5 min from the end of the stimulation, and normalized to their values assessed at the beginning of the experiment. The stimulation was performed with devices having two different heights (0.5 and 1 cm) with **(B)** or without **(A)** the palatal stimulation. ^∗∗∗^, *t*-test, *p* < 0.001.

Finally, we assessed the dependence of the analgesic effect on the level of mandibular extention by comparing the above shown results obtained with a 1 cm high device, with devices having a height reduced to 0.5 cm. These experiments were performed both for the sole proprioceptive and the proprioceptive plus exteroceptive stimulation. As shown in **Figure 5** where the PPT and PTL values at 7 min of stimulation and at 5 min from the end of the stimulation are shown, the analgesic effect obtained with the 0.5 cm devices was significantly smaller than that obtained using the 1 cm devices. This suggests that the level of mandibular extention represents a sensitive parameter that should be taken into consideration to optimize the analgesic effect.

## Discussion

In this study we found that a proprioceptive sensory stimulation of the TMJ and masticatory muscles produces an appreciable antinociceptive effect, assessed by evaluating the PPT and the PTL in response to electrical stimulation of the incisor teeth. We further found that the effect depended on the level of mandibular extension induced on the subject, and was increased when an exteroceptive stimulation of the palatal spot was added. These data are in accordance with the GCT at the trigeminal level, and open to new strategies to contrast the pain originating from the mouth, and possibly from other districts of trigeminal origin, as previously proposed (Pannain et al., 1973; Pannain and Zampino, 1980). A limitation of our study may originate from the contamination of the proprioceptive stimulus by exteroceptive activity. More specifically, in order to induce the activation of the masseter muscle proprioceptors we used a device that, in addition to producing a mandibular extension may also contact the lips, the tongue and the surrounding oral mucosa, thus inducing an additional exteroceptive stimulation.

It is known that the dental pulp contains sensory trigeminal terminals which detect pain as the only sensation, and that these nociceptors, belonging to different classes, are characterized by different activation thresholds and conduction velocities. Several studies show that nociceptive Aβ fibers, that display an activation threshold significantly lower than the Aδ and C type nociceptors, are also present in the dental pulp (Malerba, 2017). It is therefore possible that the two parameters we used to evaluate the response to pain (PPT and PTL) detect the activation of different types of pain nociceptors present in this district. In particular, the PPT, corresponding to the minimum current intensity that can be detected by the subject, could originate from the activation of the most sensitive Aβ nociceptors, while the PTL, corresponding to the maximal current intensity tolerated by the subject, could be a more unspecific parameter reporting the activation of all types of dental nociceptors (Dong et al., 1985; Trowbridge, 1986; Figdor, 1994; Niharika et al., 2013).

Previous studies have demonstrated that the electrical stimulation of the mouth leads to a significant increase in the pain threshold of the dental pulp nociceptors (Mumford, 1976; Gerschman and Giebartowski, 1991; also cf. Andersson et al., 1977). We therefore verified whether a non-electrical, less invasive trigeminal autostimulation may still have an antinociceptive effect. We found that an autostimulation obtained by simply keeping the mouth open (to produce a proprioceptive input from the masseter muscle) or by applying a small mechanical pressure to the palatal spot (producing an exteroceptive stimulation) have antinociceptive effects, which are even higher than those reported with the more invasive and unspecific electrical stimulation. Our stimulations could be certainly more easily applied to develop anesthesiology therapies.

We do not know the cellular and molecular mechanism of these effects, but certainly the first option points to a gate control in the trigeminal system, whose presence has been widely demonstrated in other body districts. In this view, proprioceptive input from masseter muscles and exteroceptive input from palatal area, resulting from the application of the mechanical devices of **Figures 1A,B**, and traveling through Aβ mechanoreceptive fibers, would increase the activity of the inhibitory interneurons connected to second order neurons, thus modulate negatively the nociceptive transmission through C fibers, and alleviate pain. Other possibilities should, however, be considered. First, it is also possible that the anti-nociceptive effect described here is a consequence of the instauration of the so-called trigemino-cardiac reflex (TCR), known to induce bradycardia and hypotension in response to a stimulation of the trigeminal nerve in patients during surgery, effects mediated by the activation of the vagus nerve (Kumada et al., 1977; Schaller, 2004; Schaller et al., 2007). Brunelli et al. (2012) have further shown that a brief (10 min) proprioceptive stimulation induced by mandibular extension is able to cause an appreciable bradycardic and hypotensive effect in humans. Subsequent studies performed in rats demonstrated that this effect required intact trigeminal nerve, suggesting that it may be considered an expression of the TCR (Lapi et al., 2013, 2014). Notably it has been shown that the activity of the vagus nerve may lead to a substantial reduction of acute pain (Kirchner et al., 2000, 2006). Another possibility is that the effect is mediated by nitric oxide (NO). Previous studies have demonstrated that a mandibular extension very similar to that applied in our subjects, was able to substantially lower the systemic blood pressure in humans, and lead to an increase in the NO plasma levels in the rat (Brunelli et al., 2012; Lapi et al., 2013, 2014, 2016, 2017; Del Seppia et al., 2016, 2017). Notably, NO has been implicated in pain transmission (Rosenthal et al., 2015), and in some cases NO donors have been shown to produce antinociceptive effects, while NO synthase inhibitors lead to pain (Yeo, 2002; da Silva et al., 2008; Fan et al., 2009). It is thus possible that an increase in the NO plasma levels produced by proprioceptive and exteroceptive stimuli may explain, at least in part, the increase in the PPT and PTL in our experiments. However, it needs to be stressed that the TCR and the increase in the NO levels may explain the proprioceptive component induced by mandibular extension, but not the antinociceptive effect of the exteroceptive palatal stimulation. Further studies are needed to fully clarify the antinociceptive mechanism of the non-painful sensory mouth stimulation.

## Ethics Statement

This study was carried out in accordance with the recommendations of the ethics committee of the University of Perugia. The protocol was approved by the ethics committee (verbale numero 2 del 20-06-2016). All subjects gave written informed consent in accordance with the Declaration of Helsinki.

## Author Contributions

CZ, MC, and RF performed the pain tests. LC performed the data analyses. LC and FF wrote the paper. All authors approved the final version of the Manuscript.

## Conflict of Interest Statement

The authors declare that the research was conducted in the absence of any commercial or financial relationships that could be construed as a potential conflict of interest. A patent (International publication number WO 2014-020483; Title: Neurophysiological stimulation device) belongs to an author of the paper (RF). There is not competing interest, as the authors do not see any compromise of objectivity or validity of the research. The reviewer BS and handling Editor declared their shared affiliation.
